# Donor-derived cell-free DNA as a diagnostic marker for kidney-allograft rejection: A systematic review and meta-analysis

**DOI:** 10.17305/bb.2024.10049

**Published:** 2024-08-01

**Authors:** Yanbo Xing, Qiang Guo, Cong Wang, Haoying Shi, Jiarui Zheng, Yijun Jia, Chengyong Li, Chuan Hao

**Affiliations:** 1 Department of Urology, The Second Hospital of Shanxi Medical University, Taiyuan, Shanxi, China; 2 Second Clinical Medical College, Shanxi Medical University, Taiyuan, Shanxi, China

**Keywords:** Donor-derived cell-free DNA (dd-cfDNA), diagnosis, kidney, meta-analysis, rejection

## Abstract

Donor-derived cell-free DNA (dd-cfDNA) has emerged as a promising biomarker for detecting graft rejection. This study aimed to evaluate the diagnostic accuracy and clinical value of applying it to kidney transplant rejection. Relevant literature on dd-cfDNA diagnostics in kidney transplant rejection was reviewed from PubMed, Embase, Cochrane Library, and Web of Science databases up to 2023. Data and study characteristics were extracted independently by two researchers. Diagnostic accuracy data for any rejection (AR) and antibody-mediated rejection (ABMR) were analyzed separately. Potential heterogeneity was analyzed by subgroup analysis or meta-regression. Funnel plots were used to clarify the presence or absence of publication bias. Nine publications provided data on dd-cfDNA accuracy in diagnosing patients with AR. The pooled sensitivity, specificity, and the area under the receiver operating characteristic (AUROC) curve with 95% confidence intervals (CIs) were 0.59 (95% CI, 0.48–0.69), 0.83 (95% CI, 0.76–0.88), and 0.80 (95% CI, 0.76–0.83), respectively. Additionally, 12 studies focused on the diagnostic accuracy of dd-cfDNA for ABMR, showing pooled sensitivity, specificity, and the AUROC curve with 95% CI of 0.81 (95% CI, 0.72–0.88), 0.80 (95% CI, 0.73–0.86), and 0.87 (95% CI, 0.84–0.90), respectively. Study type, age group, and sample size contributed to heterogeneity. In summary, our findings indicate that while plasma dd-cfDNA accuracy in diagnosing patients with AR is limited by significant heterogeneity, it is a valuable biomarker for diagnosing ABMR.

## Introduction

Organ transplantation represents a pinnacle in surgical interventions for organ failure in patients. Among these, kidney transplantation has emerged as the optimal treatment for end-stage kidney disease, owing to its rapid and pioneering advancements, alongside its well-established technology and proficient management [1]. Kidney transplantation significantly enhances the patients’ survival rate and quality of life compared to dialysis [2]. However, transplant rejection is a major factor affecting the survival of solid transplanted organs [3]. Hence, it is crucial to promptly and precisely identify graft rejection and administer efficient therapy to prolong patient survival.

Currently, there are no reliable biomarkers for identifying graft rejection. Assessing transplant kidney rejection using traditional laboratory indices, such as serum creatinine, urea nitrogen, and creatinine clearance offers limited value due to their low sensitivity and specificity [1]. Furthermore, a study has indicated that diagnosing rejection based on serum creatinine is often delayed, with approximately 50% of transplanted kidneys potentially suffering functional impairment by the time serum creatinine levels become abnormal [4]. While pathologic biopsy remains the preferred method for diagnosing rejection and stands as the gold standard, it is invasive and carries potential complications [5, 6].

Donor-derived cell-free DNA (dd-cfDNA) is a fragment of DNA released by cell death that exists in free form in the circulation and is not bound to cells. High levels of dd-cfDNA indicate graft damage and can be observed days or even weeks before the onset of acute rejection symptoms [1]. Several studies have reported the clinical validity of utilizing dd-cfDNA in kidney transplantation for the identification or exclusion of rejection or other graft injuries [7–9]. However, data from the study by Chang et al. [10] do not support its use as a biomarker of transplant rejection. Discrepancies between different studies exist, likely due to variations in research methodology, threshold setting, and study population. We conducted this meta-analysis to resolve the existing controversies and determine dd-cfDNA’s role as a diagnostic indicator for kidney transplant rejection.

## Materials and methods

This systematic review was registered in the International Prospective Register of Systematic Reviews (PROSPERO; CRD42023463407) and conducted following the PRISMA guidelines [11].

### Search strategy

We systematically searched PubMed, Web of Science, Embase, Cochrane, and various other databases for studies evaluating the accuracy of dd-cfDNA in diagnosing rejection after kidney transplantation. The heading terms included “transplantation”, “dd-cfDNA”, and “sensitivity or specificity”. Moreover, we assessed the bibliographies of selected research papers and contacted the authors when necessary. The search concluded on October 1, 2023.

### Study selection

The included studies evaluated the diagnostic accuracy of blood dd-cfDNA in detecting graft rejection. Studies of urinary dd-cfDNA were not included. Eligible studies included cohort studies, case-control studies, and cross-sectional studies. Furthermore, the research provided comprehensive data for the construction of a 2 × 2 contingency table, including information on false positives (FPs), true positives (TPs), and negatives. We did not include animal tests, evaluations, letters, case studies, professional viewpoints, or editorial pieces.

### Data extraction

The data from all included studies were independently extracted by two investigators using the predefined data extraction form. Consultation with a third reviewer resolved any arising differences. Among the extracted information were the author’s name, publication year, study design, sample size, test method, cutoff thresholds, TPs, FPs, false negative cases (FNs), true negative cases (TNs), as well as sensitivity and specificity values.

### Quality assessment

Quality Assessment of Diagnostic Accuracy Studies-2 (QUADAS-2) tool was used to assess study quality [12]. Two authors independently evaluated each signaling question, assigning a score of “yes”, “no”, or “unclear”. In case of disagreements, a third reviewer was involved for consensus.

### Statistical analysis

The bivariate approach was employed to assess combined sensitivity, specificity, positive and negative likelihood ratios (PLR and NLR), and the diagnostic odd ratio (DOR). This analysis also produced the corresponding 95% confidence interval (CI) and generated the summary receiver operating characteristic curve (sROC).

We investigated the threshold phenomenon in this study using the Spearman correlation coefficient due to the varying cutoff values utilized in the included studies. The chi-square test and *I*^2^ test were employed to assess heterogeneity in the non-threshold effect. Significant heterogeneity was defined as *I*^2^ > 50%. Subgroup analyses and meta-regression were conducted to explore sources of heterogeneity.

Deeks’ funnel plot was utilized to assess the potential presence of publication bias. Statistical analyses and meta-analyses were conducted using Stata version 15.1 (StataCorp, TX, USA).

## Results

**Figure 1.**
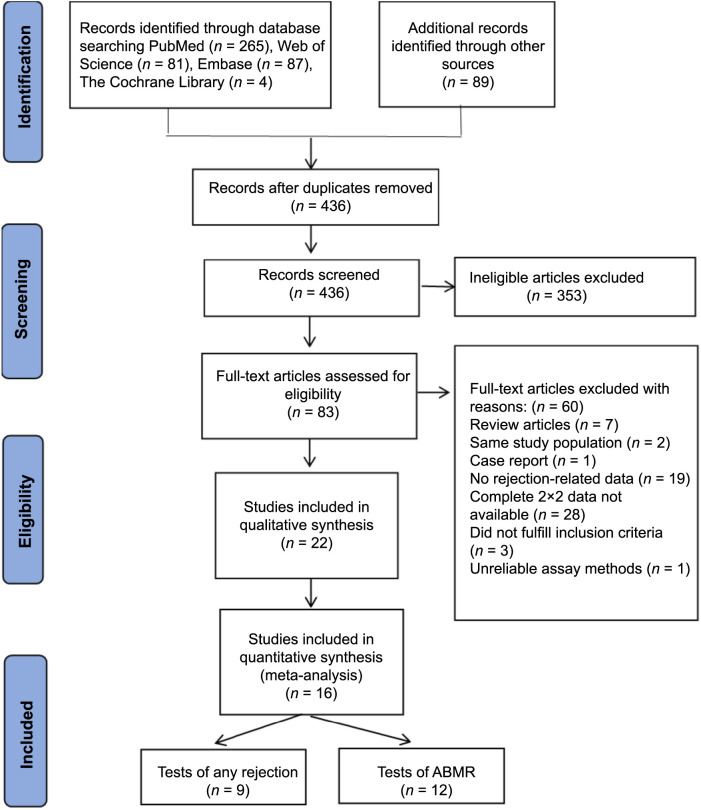
**The flow diagram of the search and selection process.** ABMR: Antibody-mediated rejection.

We initially identified 526 articles from five databases. Following the elimination of duplicates and irrelevant studies based on title and abstract screening, a total of 83 articles were chosen for a comprehensive review of the full text in order to extract pertinent information. After conducting a comprehensive analysis of the entire document, we incorporated a total of 22 articles, excluding reviews, case studies, and articles with unattainable or incomplete information (Figure 1) [7–10, 13–30]. Additionally, Gielis et al.’s [31] study was excluded due to its heavy reliance on unreliable Sequido assay methods.

### Characterization of the studies

The meta-analysis included the diagnostic criteria and distinctive features of every study, as displayed in Table 1. All studies were published within the last five years. Among these, 14 studies were conducted in the United States, 3 in China, 3 in Australia, 1 in Canada, and 1 in Germany. More than half of the studies were prospective in nature. The most widely employed method for measuring dd-cfDNA is Next-Generation Sequencing (NGS). In the majority of the included literature, AlloSure, developed and provided by CareDx, Inc., serves as a prominent NGS-based detection method for dd-cfDNA assessment. Additionally, ddPCR has also been successfully employed. The cutoff value of 1.00% was frequently employed across the majority of the studies. Diagnostic criteria for pathologic biopsies across all studies adhered to the Banff classification.

**Table 1:** Summary characteristics of included studies

**First author**	**Year**	**Study type**	**Country**	**Sample type**	**Test method**	**Reference standard**	**Rejection type**	**Threshold (%)**	**TP (%)**	**FP (%)**	**FN (%)**	**TN (%)**	**SEN (%)**	**SPE (%)**	**AUC**
Cheng	2022	Retrospective	China	Plasma	ND	Banff, 2017	ABMR	1.11	16	17	2	25	88.9	59.5	0.76
Zhang	2020	Prospective	China	Plasma	NGS	Banff, 2015	ABMR	1.00	16	5	2	14	88.9	73.7	0.9
Jordan	2018	Prospective	United States	Plasma	NGS	Banff, 2013	ABMR	1.00	13	13	3	61	81.3	82.4	0.86
Dauber	2020	Prospective	Austria	Plasma	qPCR	Banff, 2015	Acute rejection	2.70	7	4	1	17	87.5	81.0	0.84
Huang	2019	Retrospective	United States	Plasma	NGS	Banff, 2013	Any rejection	1.00	23	8	11	21	67.6	72.4	ND
							ABMR	1.00	20	11	4	28	83.3	71.8	ND
Sigdel	2018	Retrospective	United States	Plasma	mmPCR-NGS	Banff, 2017	Active rejection	1.00	33	50	5	129	86.8	72.1	0.87
Oellerich	2019	Prospective	Germany	Plasma	ddPCR	Banff, 2017	Any rejection	0.43	16	122	6	273	72.7	69.1	0.73
Whitlam	2019	Prospective	Australia	Plasma	ddPCR	Banff, 2013	ABMR	0.75	11	12	2	36	84.6	75.0	0.91
Bloom	2017	Prospective	United States	Plasma	NGS	Banff, 2013	Active rejection	1.00	16	10	11	70	59.3	87.5	0.74
							ABMR	1.00	13	15	3	76	81.3	83.5	0.87
Bunnapradist	2021	ND	United States	Plasma	mmPCR-NGS	Banff, 2017	Active rejection	1.00	7	3	2	29	77.8	90.6	ND
Park	2021	Prospective	United States	Plasma	NGS	Banff, 2019	Acute rejection	1.00	42	11	61	114	41.0	91.2	ND
Mayer(1)	2021	Retrospective	Austria	Plasma	NGS	Banff, 2017	ABMR	0.78	20	4	5	16	80.0	80.0	0.89
Mayer(2)	2021	Retrospective	Austria	Plasma	NGS	Banff, 2017	ABMR	0.62	15	6	2	7	88.2	53.8	0.69
Halloran	2022	Prospective	Canada	Plasma	NGS	Banff, 2019	Any rejection	1.00	40	20	14	75	74.1	78.9	ND
Puliyanda	2022	Prospective	United States	Plasma	NGS	Banff, 2017	Acute rejection	1.00	18	0	3	27	86.0	100.0	0.996
Obrişcă	2022	Retrospective	United States	Plasma	NGS	Banff, 2017	Any rejection	1.00	19	2	11	22	63.3	91.7	ND
							ABMR	1.00	17	4	1	32	94.4	88.9	ND
Chang	2022	Retrospective	United States	Plasma	ND	Banff, 2019	Any rejection	1.00	16	9	51	160	24.0	95.0	ND
							ABMR	1.00	11	15	13	197	45.8	92.9	ND
Cheng	2021	Retrospective	China	Plasma	ND	Banff, 2017	ABMR	0.96	19	1	2	28	90.5	96.6	0.9
Ranch	2023	Prospective	United States	Plasma	ND	Banff criteria	Any rejection	1.00	23	1	12	25	65.7	96.2	ND
								0.50	25	11	10	15	71.4	57.7	ND
Dandamudi	2022	Prospective	United States	Plasma	NGS	Banff criteria	Acute rejection	1.00	9	3	18	79	33.3	96.3	0.82
								0.50	21	18	6	64	77.8	78.0	ND
Rizvi	2023	Prospective	United States	ND	ND	Banff, 2017	Any rejection	1.00	32	8	20	29	61.5	78.4	ND
								0.50	43	16	9	21	82.7	56.8	ND
							ABMR	1.00	20	8	8	29	71.4	78.4	ND
								0.50	23	16	5	21	82.1	56.8	ND
Gupta	2022	Prospective	United States	ND	ND	Banff, 2017	Any rejection	1.00	38	18	40	112	48.7	86.2	ND
Bu	2022	Prospective	United States	ND	NGS	Banff, 2019	Any rejection	1.00	65	19	48	87	57.5	82.1	ND
								0.50	88	31	25	75	77.9	71.0	ND
							ABMR	1.00	49	36	26	108	65.3	75.0	ND
								0.50	59	59	16	85	78.7	59.0	ND

TP, FP, FN and TN are presented as absolute counts. TP: True positive; FP: False positive; FN: False negative; TN: True negative; SEN: Sensitivity; SPE: Specificity; AUC: Area under the curve; ABMR: Antibody-mediated rejection; ddPCR: Digital droplet polymerase chain reaction; NGS: Next-generation sequencing; qPCR: Quantitative polymerase chain reaction; ND: No data.

Within the included literature, nine articles are focused on identifying any rejection (AR), encompassing T cell-mediated rejection (TCMR), antibody-mediated rejection (ABMR), or mixed rejection (specific rejection types are detailed in Table S1). Additionally, 12 articles specifically assessed the identification of ABMR. Considering the distinct types of rejection, separate meta-analyses were conducted for AR and ABMR. Furthermore, a limited number of studies have individually explored the diagnostic performance of dd-cfDNA in distinguishing active rejection from acute rejection. However, due to the scarcity of literature on this specific aspect, we did not include them in the quantitative analysis.

### Quality assessment of included literature

**Figure 2.**
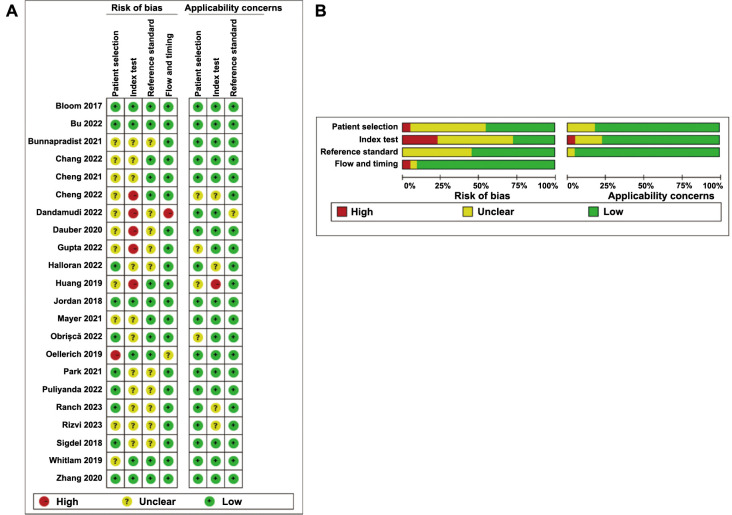
**Risk of bias analysis using QUADAS-2 tool for studies included in the meta-analysis.** (A) Risk of bias for individual studies; (B) Summary risk of bias for each domain. QUADAS-2: Quality assessment of the diagnostic accuracy studies-2.

Out of the 22 papers analyzed, 11 studies demonstrated a low risk in terms of patient selection, while the remaining studies exhibited an indication of a higher risk, primarily due to the inclusion of patients from non-randomized or non-consecutive sampling. Most studies lacked predetermined thresholds, leading to their classification as “unclear risk” in the index test domain. None of the studies included in the reference standard domain were deemed “high risk,” as all of them utilized pathologic examination (kidney puncture biopsy) to determine whether a patient had experienced rejection. The majority of studies in the flow and timing domains were assessed as having a “low risk” for bias (Figure 2).

### Diagnostic accuracy of dd-cfDNA in any rejection (AR)

**Figure 3.**
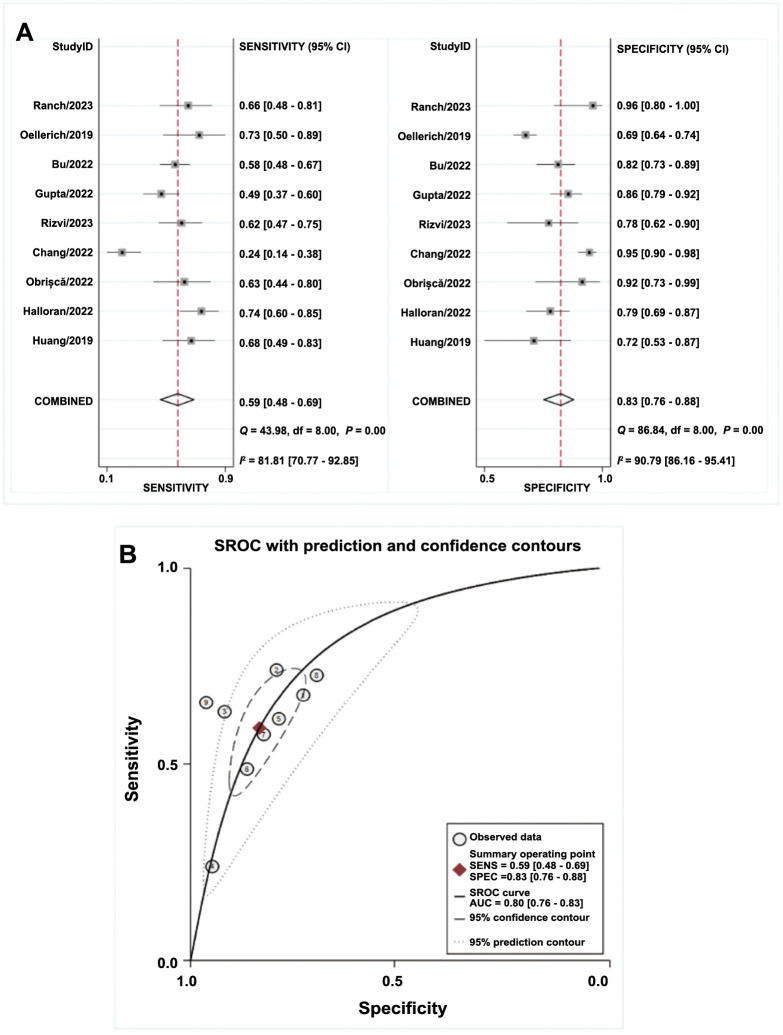
**Summary of the diagnostic accuracy of dd-cfDNA for diagnosis of AR**. (A) Pooled sensitivity and specificity; (B) sROC with prediction and confidence contours. dd-cfDNA: Donor-derived cell-free DNA; AR: Any rejection; sROC: Summary receiver operating characteristic curve; SENS: Sensitivity; SPEC: Specificity; AUC: Area under the curve; CI: Confidence interval.

Out of the studies included, nine reported the diagnostic accuracy of dd-cfDNA for AR, with one specifically focusing on children. The meta-analysis showed that the pooled sensitivity and specificity were 0.59 (95% CI, 0.48–0.69) and 0.83 (95% CI, 0.76–0.88), correspondingly (Figure 3A). The area under the curve (AUC) was 0.80 (Figure 3B), and the overall DOR was 7 (95% CI, 5–10). The pooled PLR was 3.5 (95% CI, 2.8–4.5), and the pooled NLR was 0.49 (95% CI, 0.40–0.61).

### Heterogeneity and subgroup analysis

We found significant heterogeneity in sensitivity (*P* < 0.01; *I*^2^ ═ 81.8%) and specificity (*P* < 0.01; *I*^2^ ═ 90.8%) among the included studies. The Spearman correlation of 0.517 (*P* ═ 0.15) suggests that the threshold effect did not predominantly contribute to the heterogeneity.

We conducted a subgroup analysis based on threshold levels. The subset that had a threshold of 1% exhibited greater combined specificity (0.85 compared to 0.66), while the subset with a threshold of 0.5% demonstrated higher sensitivity (0.78 compared to 0.57). Both subsets displayed similar PLR, NLR, DOR, and AUC values, as shown in Table S2.

Considering the differences between children and adults, we performed a subgroup analysis. In the adult group, our results indicate a combined sensitivity of 0.59 (95% CI, 0.46–0.70), a combined specificity of 0.83 (95% CI, 0.75–0.88), and an AUC of 0.79 (95% CI, 0.75–0.82) (Table S2). Due to the limited number of studies in the pediatric group, we refrained from conducting a quantitative analysis on it.

Upon reviewing the experimental designs in the literature, we observed that some subjects underwent protocol biopsies, while others underwent “clinically indicated” biopsies based on direct evidence of graft damage in the patient, such as elevated baseline serum creatinine and/or new-onset of proteinuria. The subgroup analyses showed that the protocol-contained group and the for-cause group had comparable pooled sensitivity and specificity when compared to the overall group (Table S2).

To delve deeper into the origins of heterogeneity, we performed meta-regression on the chosen covariates. The main covariates included dd-cfDNA detection methods, biopsy design, type of study, study size, and age groups. The results showed that the study type (*P* < 0.01), sample size (*P* < 0.001), and age group (*P* < 0.001) contributed to heterogeneity (Figure S1).

Ultimately, Deeks’ funnel plot asymmetry test was executed to assess publication bias. Findings indicated that there is no publication bias (*P* ═ 0.11) (Figure S4A).

### Diagnostic accuracy analysis of dd-cfDNA in antibody-mediated rejection (AMBR)

**Figure 4.**
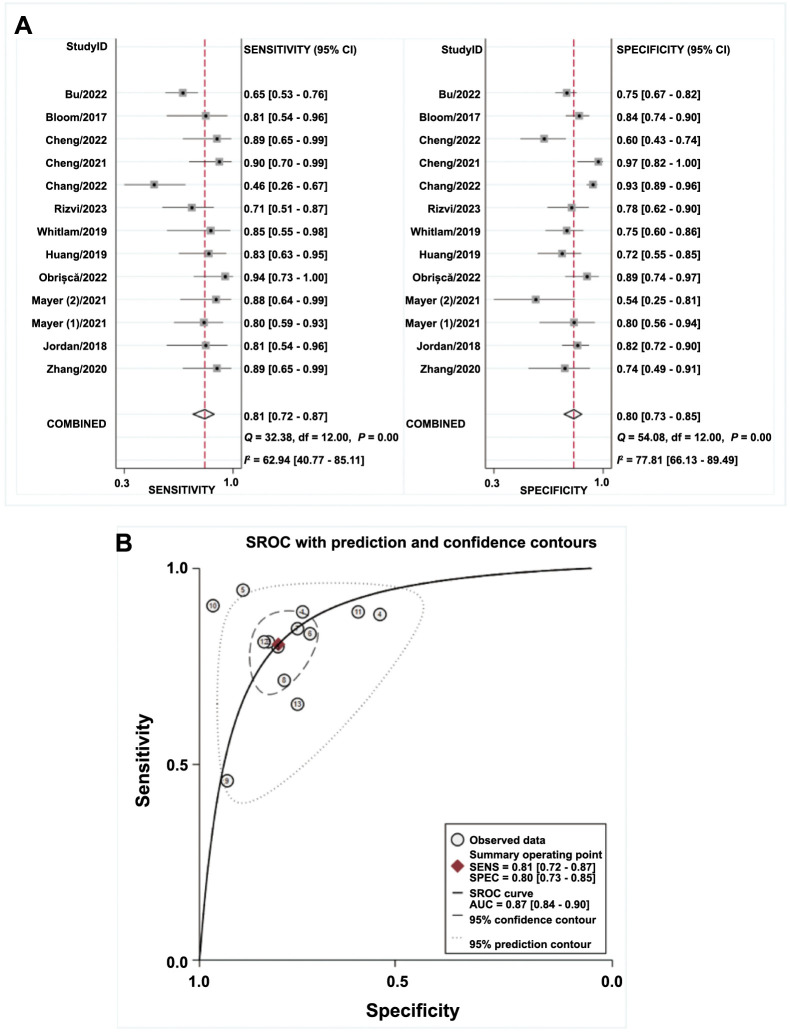
**Summary of the diagnostic accuracy of dd-cfDNA for diagnosis of ABMR.** (A) Pooled sensitivity and specificity; (B) sROC with prediction and confidence contours. dd-cfDNA: Donor-derived cell-free DNA; ABMR: Antibody-mediated rejection; sROC: Summary receiver operating characteristic curve; SENS: Sensitivity; SPEC: Specificity; AUC: Area under the curve; CI: Confidence interval.

The meta-analysis encompassed 12 diagnostic tests to evaluate the accuracy of diagnosing ABMR. Mayer’s article reports on two different sets of data and is considered two separate studies. Forest plots illustrate the sensitivity and specificity in Figure 4A. The overall sensitivity was 0.81 (95% CI, 0.72–0.87), and specificity was 0.80 (95% CI, 0.73–0.85), respectively. The pooled estimates for PLR, NLR, and DOR were 4.0 (95% CI, 3.0–5.3), 0.24 (95% CI, 0.17–0.35), and 17 (95% CI, 10–28), respectively. The AUC value was 0.87, indicating a high diagnostic accuracy for ABMR using dd-cfDNA.

### Test of heterogeneity and subgroup analysis

Variation among studies was observed regarding sensitivity (*P* < 0.01; *I*^2^ ═ 65.8%) and specificity (*P* < 0.01; *I*^2^ ═ 79.7%). The Spearman correlation of 0.098 (*P* ═ 0.761) suggests that the threshold effect did not significantly contribute to the heterogeneity.

Moreover, when considering only prospective studies, the combined sensitivity and specificity of the prospective study group were 0.76 (95% CI, 0.66–0.84) and 0.79 (95% CI, 0.74–0.83), respectively (Table S3). No heterogeneity was observed in sensitivity (*P* ═ 0.23; *I*^2^ ═ 28.0%) and specificity (*P* ═ 0.58; *I*^2^ ═ 0) among prospective studies (Figure S2).

When limiting the analysis to the literature on the for-cause group, the summary estimate showed a sensitivity of 0.83 (95% CI, 0.74–0.89) and a specificity of 0.80 (95% CI, 0.72–0.86), which were consistent with the overall findings (Table S3).

Similarly, our meta-regression results reveal that study type (*P* < 0.05), testing method (*P* < 0.01), and sample size (*P* < 0.001) significantly contribute to the observed heterogeneity (Figure S3). Additionally, Deeks’ funnel plot analysis showed no substantial indication of publication bias (Figure S4B).

## Discussion

Over the past five years, several organizations have assessed the potential of dd-cfDNA as a biomarker for graft rejection in transplants, including the heart, liver, and kidney. The results of our meta-analysis suggest that dd-cfDNA can be a helpful marker for identifying ABMR. However, further validation is still required to assess its accuracy in recognizing AR. In addition, a few studies have investigated the diagnostic performance of dd-cfDNA in distinguishing acute rejection from active rejection. However, due to the limited number of relevant studies, a Meta-analysis was not conducted.

In comparison to dd-cfDNA, serum creatinine demonstrates neither specificity nor sensitivity in recognizing allograft injury. A previous study indicated that at the time of biopsy, serum creatinine failed to discriminate active rejection from no active rejection. The ROC curve for creatinine in distinguishing active rejection had an AUC of 0.54, indicating that at any cutoff level for creatinine, there were as many false positives as true positives [7]. In contrast, our meta-analysis found that dd-cfDNA exhibited moderate sensitivity and high specificity. Moreover, it demonstrated a high level of diagnostic accuracy in predicting patients with AR, with a combined AUC of 0.80.

Currently, there is a lack of direct comparative research on the diagnostic performance of dd-cfDNA testing methods applied to pediatric and adult transplant groups. In our study, when the dd-cfDNA threshold was set at 1% to identify adult AR, the AUC was 0.79, with a specificity of 83% and a sensitivity of 59%. However, due to limited data on pediatric patients, we are unable to conduct quantitative analysis in this regard. Findings from Ranch et al.’s [24] study in the pediatric group indicated that when dd-cfDNA>1%, the sensitivity for diagnosing AR is 66%, with a specificity of 96%.

Puliyanda posited that there are differences in the sensitivity and specificity of dd-cfDNA between pediatric and adult patients, and these variations may be attributed to the difference in size between the graft and the recipient’s body [14]. The lower body weight of children, resulting in reduced background DNA, is also a contributing factor [14]. Additionally, a study highlighted that due to the more significant kidney reserve provided by adult-sized grafts, serum creatinine in pediatric recipients remained within the normal range even during rejection [32]. In this context, the role of dd-cfDNA becomes particularly crucial in identifying rejection in children, given its high specificity, thereby significantly reducing the need for unnecessary biopsies.

The diversity of thresholds reflects the fact that centers are still in the exploratory phase, and currently, there are no uniform laboratory standards. When we used the threshold published in most studies (1%) as a criterion for the diagnosis of rejection, our study yielded an AUC of 0.81 and a diagnostic specificity of 0.85. In contrast, the sensitivity was lower, at 0.57, and was prone to FN results. Notably, the subgroup with a threshold ═ 0.5% exhibited a higher sensitivity of 0.78, although the specificity was reduced to 0.66. Hence, additional research is required in the coming years to ascertain the most suitable threshold for dd-cfDNA.

Although we performed separate subgroup analyses based on threshold and reason for biopsy, the results of these analyses showed small deviations in effect sizes for diagnostic metrics compared with the overall results. To investigate the origins of variability more deeply, we conducted a meta-regression, revealing that both study type and sample size contributed to the observed heterogeneity. Yet, due to limited validated data, further stratification of the included patients in the study was unattainable.

In this study, we observed that the combined sensitivity of dd-cfDNA in detecting ABMR patients is greater compared to its sensitivity in detecting patients with AR, despite having the same specificity. This pattern remained consistent when excluding studies with higher heterogeneity, specifically retrospective studies. The statistical difference in sensitivity could potentially be attributed to the inclusion of patients with TCMR in the study. Specifically, when ABMR occurs, it involves interactions between antibodies and the endothelium of allograft vessels, leading to the release of dd-cfDNA directly into the bloodstream. In contrast, the concentration of dd-cfDNA is correlated with the grade of TCMR, where borderline and grade 1A TCMR involve fewer vessels, resulting in mostly normal concentrations of dd-cfDNA.

A further reason for false negative TCMR results may be attributed to the use of relatively long amplicons (100–130 bp) in some NGS methods [3, 7, 18]. In TCMR there may be more extensive dd-cfDNA fragment degradation. Dauber et al. [15] demonstrated significantly higher dd-cfDNA test results when using smaller-size amplicons. A range of 66–103 bp has recently been recommended [33]. However, due to the limited availability of raw data, we were unable to conduct a separate quantitative analysis of TCMR patients. Further research is needed in the future to explore the diagnostic performance of NGS testing for detecting TCMR.

In an effort to further enhance the diagnostic performance of dd-cfDNA, researchers have made various attempts. Mayer’s study suggests that, in comparison to serum creatinine, dd-cfDNA can be utilized for the early detection of ABMR in kidney transplant patients who are positive for donor-specific antibodies (DSA). Furthermore, the combined detection of dd-cfDNA and DSA enhances diagnostic performance [19]. Additionally, a few articles have explored the potential of quantifying absolute dd-cfDNA (copies/ml) as an alternative method. Two prior investigations yielded conflicting findings regarding the absolute values of dd-cfDNA [21, 26]. Oellerich et al. highlighted the superiority of absolute dd-cfDNA quantification over dd-cfDNA fraction for distinguishing kidney allograft rejection. They asserted that absolute dd-cfDNA values remain unaffected by alterations in circulating recipient DNA, rendering them more dependable [21]. Nevertheless, these findings were not substantiated in the study conducted by Whitlam et al. [26]. This discrepancy seems to be due to methodological differences, as Whitlam did not account for variation in cfDNA extraction efficiency and ddPCR amplification. These two variables account for 44%–55% of the underestimation of the number of DNA copies per ml. In a study by Bunnapradist et al. [13], it was demonstrated that incorporating both the quantity and percentage of dd-cfDNA enhanced the test’s sensitivity in kidney transplant patients while maintaining a high level of specificity compared to using only the dd-cfDNA percentage. Nevertheless, further studies are necessary to confirm the clinical significance of absolute dd-cfDNA levels due to the limited sample size [34].

There are some limitations to our study. Firstly, despite the implementation of stringent inclusion criteria, heterogeneity could not be entirely eliminated. Secondly, due to analytical challenges associated with some NGS methods for TCMR detection, the overall diagnostic value of dd-cfDNA seems not to be correctly represented by any AR in the presented systematic review. Thirdly, after a thorough review of the included literature, we found that, due to limited available information, specific diagnostic accuracy data for dd-cfDNA in distinguishing TCMR could not be obtained.

## Conclusion

In conclusion, although the accuracy of Plasma dd-cfDNA in diagnosing patients with AR is not very reliable due to the observed large heterogeneity, our findings affirm the utility of Plasma dd-cfDNA levels as a diagnostic indicator for ABMR. However, there remains a necessity for future multicenter, prospective studies to investigate the ideal threshold of dd-cfDNA% for diagnosing rejection. Moreover, exploring the potential simultaneous use of dd-cfDNA quantification to enhance diagnostic accuracy is warranted.

## Supplemental data

Supplementary data can be found at the following link: https://www.bjbms.org/ojs/index.php/bjbms/article/view/10049/3153
